# Immigrant women’s diet challenges and strategies to achieve healthy eating behaviour for oneself and one’s family – a qualitative story dialogue method study from Norwegian context

**DOI:** 10.1186/s40795-026-01412-2

**Published:** 2026-07-03

**Authors:** Nima Neolene Wesseltoft-Rao, Usha Subramanian, Jemimah Yambo Sigurdsen, Else Storaas Vatne, Kristin Åmli Van Gilst, Zada Pajalic

**Affiliations:** 1 Faculty of Health Sciences, Sustainable Health Care and Welfare Technologies (SHWT) Research Group, VID Specialized University, Post-box 184, Vinderen, Oslo, 0319 Norway; 2 Christian Intercultural Work (KIA), Oslo, Norway; 3 Oslo Municipality, Oslo, Norway; 4 The Norwegian Mission Society, Oslo, Norway; 5 Nesodden Municipality, Nesodden, Norway

**Keywords:** Immigrant women, Norwegian language course, Health, Weight, Participatory research, Story-dialogue method (SDM), Empowerment

## Abstract

Immigrant women are sometimes confused about dietary advice in the Norwegian healthcare context, which is incongruent with their original traditional beliefs and food culture. This confusion increases their risk of developing food intake-related lifestyle syndromes. However, little is known about their experiences and thoughts about maintaining a healthy eating behaviour. The aim of the present study was to gain insight into how immigrant women, after participating in a nutrition course, reflect on their responsibility for their own health and weight management.

The present study has a qualitative design and a participatory action research approach. The story-dialogue method (SDM) was adopted to collect and analyse data. Immigrant women (n = 20) from six countries: Kurdistan, India, Ethiopia, Sudan, Kongo and China participated in the study.

Our study showed that the participants were motivated to take control of their diets to overcome obesity. When motivated to change their habits, the women searched for nutrition information online and sought guidance. When motivated to change, the women actively encouraged family members to switch to healthier eating habits.

The study underscores the urgency of providing culturally sensitive nutrition guidance. Motivation to change one’s eating habits and lifestyle to be healthier is necessary to achieve change. Culture sensitive nutrition guidance is not just beneficial, but essential for nutrition education and health promotion in an immigrant population. It is crucial that culturally sensitive nutrition information is easily available both online and in health care settings, to ensure the health and well-being of immigrant women and their families.

## Introduction

A healthy and varied diet high in dietary fibre and low in saturated fat, salt and sugar is recommended along with physical activity to prevent obesity and chronic diseases [1, 2]. However, dietary recommendations are not always followed [3]. One factor that influences diets are ethnic backgrounds. Studies have shown longitudinal changes from traditional diets towards mixed food habits with high levels of fat, salt and sugars in immigrant populations [4, 5]. A healthy eating behaviour is not always the main focus of traditional believes of how to maintain health [6], which may continue to influence women who immigrate to Western counties [7, 8]. Dietary habits differ considerably among ethnic groups in Western counties [9, 10]. Women often support their families’ dietary behaviours, however, country of birth, length of stay in a country, language fluency, and ethnicity are indicators for health literacy and health among women and their families [11–13]. Health promotion is often influenced by families and social networks [14].

A systematic review reviled that some immigrant women are a vulnerable within Norwegian healthcare [15]. Studies indicate that they get limited dietary advice and appear to be confused about dietary advice that is incongruent with their traditional beliefs and food culture [1, 10, 16]. Culturally sensitive nutrition education can promote communication about food and health at an early phase of the migration process and facilitate the transition to a new food environment [17, 18]. Several studies in Norway have shown that immigrant women are at risk of becoming overweight [19] and developing lifestyle disorders like type 2 diabetes [20] and cardiovascular disease [21]; however, little is known about immigrant women's own experiences about maintaining a healthy lifestyle for themselves.

The aim of the present study was to gain insight into how a proportion of immigrant women in Norway, after participating in a nutrition course, reflect on their responsibility for their own health and weight management. The results from this study can be used as a means to support immigrant women’s health in the future.

## Method

This study had a qualitative design based on the Story-dialogue method (SDM), which is built on a structured dialogue [22]. A participatory action research approach was also partially used as the participants were motivated to create action-oriented solutions for the future [23, 24].

### Study context

The study was conducted in a Norwegian language course at Christian Intercultural Work (KIA). Since 2017, KIA has offered Norwegian language and social studies courses to immigrant women who have used up their allowance of Norwegian courses through the public sector or do not have the right to receive Norwegian education. The leader of KIA (ESV) and the leader of the Norwegian courses (KÅVG) are both co-authors of this article. The women attending KIA courses represented various nationalities, ages, education levels, work experiences and socio-economic backgrounds.

In addition, KIA has several activities outside of course time, in order to allow women to meet in an informal social arena, contact volunteers and practise speaking Norwegian. KIA experience that many immigrant women have diffuse health problems and that they don’t take part in existing training and welfare services like the rest of the population. Few of the women participating in KIA are physically active, and reported that they sit still a lot. Thus, KIA developed a project called “Contribution to Safety and Good Health for Minority Women”, offering training and guidance in relation to health issues and diet. Women were able to participate regardless of social, linguistic and economic status. The project aimed to help participating women become more confident, with a greater degree of mastery of the Norwegian vocabulary, when dealing with the Norwegian healthcare system and authorities. Furthermore, one of the programme’s goals was to provide participants with knowledge and practical advice about nutrition.

### Participants

All women attending a nutrition course were invited to participate in a facilitated workshop inspired by the SDM on a separate date. Six women (32–44 years) volunteered to participate in the study. The number of participants were considered sufficient as this is an explorative qualitative study. The participants were from six countries: Kurdistan, India, Ethiopia, Sudan, Kongo and China. They had lived in Norway for one to 11 years and had one to four children. Some of them worked, including in a kindergarten and a pharmaceutical company, and some were students who had an internship or were planning to apply for jobs. Their educational level varied from preparatory adult education to bachelor’s and master’s degrees.

Prior to the SDM workshop, the women were assigned to do a self-interview, reflect on the statement “My diet, my responsibility,” and prepare a story related to this specific topic.

### Nutrition course

As part of the project “Contribution to Safety and Good Health for Minority Women”, a clinical nutritionist, who is also one of the authors of this article (NWR), was engaged to lead a nutrition course. The course consisted of two classes totalling six hours about nutrition based on the NNR, including information about a varied diet with vegetables, fruit and berries, whole-grain carbohydrates, fish, lean dairy and meat products, and to limit salt and sugars, in addition to at least 30 min of activity every day [2]. The class also included practical guidance about food products from the grocery store, such as how to choose breakfast cereals that contain less sugar and more fiber, or yoghurts with less sugar and fat. The course was inspired by the nutrition education resource Healthy Start, developed for use in the Introduction Programme for refugees [18]. The Healthy Start resource is based on the Nordic Nutrition Recommendations** (**NNR), adapted to different cultures in Norway [2]. The NNR emphazise highly nutritious foods, and are associated with lower risks of cardiovascular disease and diabetes. Adopting this diet can improve metabolic health, and was assesed to be suitable for the study population [1, 2]. The classes were designed to be simple and comprehensible in Norwegian. Time was set aside for questions and conversations so that the women could help each other to put into words the issues and challenges they faced related to the theme.

Participants in the nutrition course were adult women, with an immigrant background who were born and raised in a country other than Norway and who attended Norwegian training at KIA in Oslo. Recruitment to the nutrition course took place in the spring of 2022 among immigrated women who could speak and write Norwegian. Approximately 20 women (32–44 years old) participated in the nutrition course.

### Data collection

The data collection in this study was inspired by the SDM [24, 25]. The SDM is a structured data collection and analysis method. The data was collected by two facilitators; the facilitated first author and (NWR) and supported by a skilled facilitator (ZP) in the workshop. The trigger for data collection was the participant’s “My diet, my responsibility” stories. The SDM workshop included five steps (Table 1). The session lasted for two hours.

**Table 1:** Overview of the steps, activities and questions asked inspired by Pajalic et al., 2023 [26]

Step	Activities and questions
Step 1	Introduction Introduction to the workshop procedure and the SDM steps. Participants were introduced to each other and asked to tell their story
Step 2	Reflection circle How do I relate to the story I just heard? How is this story similar to my story about planning my diet? How does the story differ from my own story? Why was my own story related to the others? What made me pick up this specific story?
Step 3	Structured dialogue
	Description phase (what questions)
	What was the identified problem regarding the topic “My diet, my responsibility”? What did I do and how did I do it? What was successful, and what did I learn in this phase? What were the challenges?
	Explanatory phase (why questions)
	Why did I choose this particular story? Why did it happen? Why did I do what I did? Why did it work? Why did it not work?
	Synthesis phase (so what questions)
	So what did I learn from this discussion? So what remains unanswered? Did I find out something unexpected while reflecting on these stories? So what is my standpoint regarding food and health? How did people or relationships change in this process?
	Action phase (now what questions)
	Now what would I do different next time? Now what would be my next step of action? What power do I have to do things differently next time? Now what are the key lessons? Now what, how can I increase my empowerment in the future?
Step 4	Story record Sharing of reflections
Step 5	Creation of insight cards Four to eight most important insights, keywords, or sentences that best summarize the dialogue are recorded

Step one consisted of an introduction to the workshop procedure. Participants were introduced to each other and asked to share their stories. The storyteller told her story based on the topic “My diet, my responsibility”. The story listener listened actively to the story, writing down notes at the end. The story recorder wrote down notes while the storyteller was talking. For each of the five steps of the SDM, the participants took all the roles (storyteller, story-listener, and story-recorder), one at a time. The Facilitator did not swich roles but stayed the same during the whole SDM process. Participants were asked to write down reflection notes on coloured post it-notes, in the form of what was called story- and insight cards [24].

Step two consists of the reflection circle. Participants were asked to reflect on the stories that were shared. The reflections were facilitated with the following questions: How do I relate to the story I just heard? How is this story similar to my story about planning my diet? How does the story differ from my own story? Why was my own story related to the others? What made me pick up this specific story?

Step three is the structured dialogue, which had four phases. The phases consisted of certain questions to encourage the reflection (Table 1): phase 1) description phase: the participants shared their stories about *what* they experienced regarding the chosen topic “My diet, my responsibility”, phase 2) explanation phase: the participants reflected on *why* they experienced what they experienced, phase 3) systemization phase: *so what* questions were asked in order to get an in-depth understanding of why they shared this particular story and phase 4) action phase: *now what* questions were asked about concrete actions that the participants could take to improve their situation related to the chosen topic.

In step four the participants share their own reflections around the stories that were told, and the story was recorded. Finally, in step five, four to eight of the most important insights, keywords, or sentences that best summarize the dialogue were recorded. In step five the final insight cards were created. Knowledge generated from the story- and insight cards were the analysis of the story dialogue and the results of the SDM session [24].

The workshop intended to go through the SDM steps, to extract the main issues regarding women’s diet, including how they understand their food intake and its relation to health.

### Analysis and reflections

#### Structured dialogue: description phase

All the workshop participants were asked to reflect on their own responsibility for their diet and share their story with the participants in the workshop. In this step study participants described and identified the problem by asking “what” they experienced related to the chosen theme.

One of the participants noticed that after her first pregnancy, she gained weight. She wasn’t aware of healthy eating habits. The elders in her family advised her on traditional methods, namely eating for two persons during pregnancy, which increased weight of 25 kilograms (kg) by the end of pregnancy. She had trouble breathing due to the weight gain. She wasn’t able to reduce her weight before her second pregnancy, which happened quickly after the first. After her second birth, she began searching the Internet for information on weight reduction methods in her mother tongue. She came across many videos and learned about healthy eating habits, including increasing the intake of vegetables and fruits in her diet. She managed to lose about 16 kg. After a gap of six years, she had a third child and managed to maintain her weight throughout the pregnancy. She ensured she was eating healthily and drinking plenty of water, even after childbirth. She consciously avoided sugar intake. She learned about intermittent fasting and began following it, which helped her to lose another 16 kg. Now the participant has good control of her weight and food intake. She has an overweight son, which is her main motivation to change the family’s lifestyle, i.e., healthy eating behaviour and going for regular walks. She takes responsibility for her own and her family’s health and diet.

The second participant mentioned that she had always been lean. At the age of 30, she had her first pregnancy and noticed hormonal changes in her body. She was also diagnosed with gestational diabetes, which meant that she had to be cautious with her sugar and food intake. Still, she gained 20 kg. After giving birth, it wasn’t easy to lose weight. She was told to eat twice as much when breastfeeding, which led to even more weight gain. Eight months after giving birth, she began to think about her body. She felt that she did not have a routine for regular food intake. She searched online and found exercise tips, but it was difficult to exercise alone. She was told that eight hours of sleep helps control food intake, and that exercise and dietary changes must be introduced simultaneously.

The third participant mentioned that she had always been lean but gained 15 kg during her first pregnancy. She was inactive and ate large portion sizes as pregnant women are usually advised to do in her culture. She drank sugary drinks whenever she felt stressed. She quickly became pregnant again and gained additional weight. This made her anxious and led her to look for information on the internet about ways to lose weight and found that large amounts of food and high intake of sugary drinks leads to weight gain. She learned that eating all the time is unhealthy. She explored weight reduction using fasting. She began with an eight-hour fasting period and gradually increased it to 24 h and included larger amounts of vegetables and water. She also adopted a few variations to the diet by eating white meat and excluding cookies and liquid sugar. She reported that drinking vinegar and regularly getting eight hours of sleep yielded some positive results.

The fourth participant said she had always been lean and managed to maintain her weight. She is conscious of her food intake. She doesn’t eat sugar, but she eats fruit. However, she gained about eight kg in weight after receiving a contraceptive injection.

The fifth participant was diagnosed with gestational diabetes during her third pregnancy. She received advice on how to control her food intake and check her blood sugar level regularly. She immediately stopped eating rice. She also introduced and adapted a new dietary pattern, consuming fish and vegetables four times a week and avoiding meat. When one of her sons became overweight, she attended a course on nutrition. She introduced everything she learned from the course, which exhibited positive results. She searched the Internet relentlessly for information about healthy food and good dietary practices. She learned about the ill effects of empty calories from sugar and stopped using it wherever possible, thereby eliminating it from her diet. The sixth participant did not share a story.

## Structured dialogue: explanatory phase

After each of the participants narrated their stories, the participants tried to understand *why* they experienced what they experienced. All participants recognized themselves and their nutritional problems based on their shared stories. Most of the participants had gained weight after pregnancy. Everyone searched for information online about diet and ways to lose weight. Subsequently, study participants made a conscious decision to change their diet.

### Weight gain after pregnancy, motivation, and strategies to lose weight

One of the participants explained that her weight increased rapidly during her pregnancies, and that her pregnancies were spaced closely together. As a pregnant woman, she felt hungry, had cravings and felt like eating all the time. After the pregnancy, she started investing in her body and did everything to lose weight. She began to eat a variety of vegetables, drink water and avoid red meat. Despite all these efforts/sacrifices, she did not lose weight to the extent she wished. Another participant reflected on the difficulty of maintaining weight in connection with the use of hormonal contraceptives.

Gestational diabetes made another participant mindful of her food intake. She noted that her son was also becoming overweight. This was one reason she wanted to focus on both of their weight problems. By searching online, she got relevant information about diet and the importance of weight management through regular activities such as walking. She changed both their diets and insisted on introducing activities in the form of walks to combat the weight gain, which gave results. Another participant’s anecdote revealed that she was often advised to eat twice as much since she was breastfeeding, which led her to rapidly gain weight that was not easy to lose. She started actively searching for information online about how to lose weight. By introducing a simple modification in her diet, which was to eliminate consumption of sugar and sweetened beverages. These modifications led to her losing 17 kg. Another participant had a similar experience when she gained 20 kg while pregnant. She decided to lose weight, believing it would be easier for her to do so if she switched to a vegetarian diet. Another participant reflected that she gained control over her weight when she introduced intermittent fasting. She began eating healthy foods, became physically active and was sure to get at least eight hours of sleep. She is still conscientious about her food choices and vigilant about her weight.

## Structured dialogue: synthesis phase

In this step the participants were asked “so what” questions to get an in-depth understanding of why study participants shared what they experienced. This step involves synthesising experiences.

### Challenges of weight loss

One of the challenges reported was that it was hard to change eating habits or strictly adhere to a healthy eating behaviour while seeing family members consuming unhealthy foods. For weight loss efforts to be successful, everyone in the family should be motivated to accept changes towards a healthy eating behaviour. It is laborious and time consuming to prepare different dishes for everyone in the family. Getting a spouse to change their food habits is a challenging task, yet some participants had succeeded on a small scale. Losing weight, or simply making weight loss efforts, is difficult without family support. If one is alone, it is easy to give up.

Participants spoke about the challenges they faced losing weight especially coming from different cultures with different habits. It is not easy to follow a rigid weight-loss programme. Another participant could relate to her gestational diabetes and the difficulties she encountered as it is not easy to lose weight when eating a diet where rice is the staple food. She had to follow a strict diet as the doctor cautioned about the impact of insulin on her baby’s growth. It was important to monitor blood sugar levels regularly. Another participant had a different view on diet plans for losing weight. She felt it was difficult to get thin by following a suggested weight-loss programme. The participant explained that one cannot lose weight only by adopting to one diet pattern. She further shared that she had searched and read several articles and was aware that diet and exercise go hand in hand: it helps if one implements both. One of the participants mentioned that some people have genes that do not respond to dietary changes. Not everyone’s body is the same, and no “one programme” works for everyone. It is important to know our body and listen and follow it.

Another point that was raised was that healthy food, such as vegetables, is expensive whereas rice is cheap. Making healthy food choices to plan a menu is a costly affair. Despite these challenges, it was considered possible to gain control over one’s food intake, physical activity, and sleep, and eat a moderately healthy diet. One participant started to run regularly and experienced that this was an effective way of maintaining her weight.

### Story dialogue: action phase

When asked “Now what?”, all participants agreed that they intended to continue their good habits and maintain a slim figure in the future. The study participants considered it essential to continue with the lifestyle changes they had implemented and saw it as a lifelong task. They said that it is important to plan out meals for the whole day and to spend at least 30 min per day on physical activity. The study participants also said that they can eat everything but in controlled amounts. They also agreed that it was important to not give up if they began to gain weight again.

## Sharing reflections

In step four of the story dialogue method, the participants shared their reflections around the stories that were shared.

### Success factors

The most crucial factors for success when changing their dietary and activity habits were considered to be a “feel good factor”. The participants stated that they felt better and confident when they weren’t overweight. The study participants mentioned that healthy food is fundamental to have good health and to keep blood sugar under control. Some had experience while others did not know ways to stop or cut down on eating unhealthy foods. All participants agreed that they must take care of their bodies as it could have repercussions later with health complications arising from unhealthy diets or inactivity. The participants expressed that they did not want to have heart disease or to be unable to use their body as before. Many of the participants turned to each other with questions. *“How do you lose weight”? “What difficulties do you face”?* One of the participants said, *“someone told me I was so fat, and it hurt me but also motivated me to lose weight. I learned that you not only have to eat healthily but also exercise a lot”*. One of the participants reflected on the significant change in friends’ reactions and comments such as *“you were thin before, you have become big now” and “Why are you so fat? Are you pregnant?”* and mentioned that it had affected her psychologically. She didn’t feel good when she heard this and thought that she needed to do something about her weight, like exercise and go on a diet.

Many participants agreed that there is pressure in society on women to be thin, they will always be judged by others regarding their weight, and that they are affected psychologically by this. The study participants said that it is hard to buy clothes if you’re overweight. One can get comments like *“it doesn’t suit you”.* Those who are close to them can make negative comments. However, on the other hand, they may wonder if something is wrong if the person loses weight. One participant reported that she lost a lot of hair and had menstrual disturbances when she gained weight. She received positive comments after losing weight and felt that she motivated others to lose weight. Finally, several participants expressed that if one gets tired of dieting, one must learn be happy with oneself and work on that.

### Sence of responsibility for one’s own health and the health of the family

The participants expressed that the body is constantly changing due to age and hormonal factors, and that age takes a toll on the body, especially after pregnancy. They agreed that when you reach your 40 s, your body is not the same as when you were in your 20 s. Many of the participants said that they want to live longer without getting sick. The study participants want to have a better lifestyle because it is essential to be strong in old age and to be able to take care of oneself. They agreed that the body takes care of you when you get old. One participant said: *“When I get old, I don’t want anyone to help me. I don’t want to be dependent on other people. My grandma was strong. When she became bedridden, she was dependent on others, and she lost respect. She was ignored. Therefore, I think it is vital to be strong and in good shape. Work now and be strong in the future”.*

To achieve this goal, the study participants expressed that it was important to avoid gaining excess weight, as well as putting off dealing with excess weight. Most of the participants want good health to care for their families and their families’ health. They spoke of the importance of teaching children to eat vegetables and of being a role model for their children. All participants mentioned that it is important to think positively, eat healthy, have healthy lifestyle habits with activities and good sleep because children learn a lot from them. Children also notice parents’ attitudes towards their own bodies, which underscores the importance of keeping a positive focus on the body.

## Results

In step five, insight cards were created to indicate concrete actions that the participants could take towards improving their situation related to the chosen theme of the SDM.

All participants recognized themselves and their nutritional problems in the shared stories. Most of the participants had gained weight after pregnancy and had taken the initiative to manage their weight by beginning to search for information online. The predominant approach reported by the participants was to find information about healthy eating and exercise and to get information online. Although each story was different, all the stories narrated had a common theme of losing weight. Participants made a conscious decision to change their diet by reducing their calorie intake, for example by limiting their intake of fat, sugar and sweetened beverages. Simultaneously, they increased their intake of water and several members introduced intermittent fasting for between eight and twelve hours. The study participants also became more observant of their sleeping habits, which was thought to have a significant impact on hormonal balance and weight loss management. Some of them started exercising regularly. One of the participants expressed, *“When I lose weight, I feel that my whole body is happy and healthy”*.

One participant summed it up this way:

*”So all of us have tried to find answers on the Internet and got a lot of information and worked with it. I don’t see much difference between us. It’s just how much you can tolerate cutting out sugar or unhealthy food and going for a walk. It is very beneficial and will help you lose weight faster than if you are inactive. So it varies from person to person. Some are skinny others become overweight. One participant here talks about how she has taken care of herself after developing diabetes. I think she is a brilliant lady, and it is amazing to take a course because you learn a lot there”.*

In the insight cards, participants agreed to highlight the following points:

1. Continue with the low-sugar diet, lots of water, and lots of vegetables. The study participants underlined that this regime feels like it’s right for the body, and it makes them feel good.
2. Being active was considered crucial. The study participants considered physical activity and going for walks outside as essential in order to get sunlight and vitamin D.
3. Furthermore, it was considered crucial to avoid stress and too many heavy thoughts. The study participants said that they as mothers had to be strong and be good role models for their children.

Other important actions mentioned to keep the motivation to maintain a healthy eating behaviour and lifestyle were to:

4. think positive
5. read and learn something new
6. listen to music
7. socialize
8. meditation

Thematic insights were drawn from the story dialogue notes and synthesized by group-based reflections and analysis in step three. Two of the researchers (US and NWR) synthesised the data.

## Discussion

The results indicate that all participants were motivated to take control of their diet to overcome obesity. The study participants had a common strategy of searching for information on the Internet and actively participating in the nutrition course and the workshop. Our results are confirmed by another study which showed that women have a positive attitude towards changing their eating habits [27]. Another study from Norway showed that most women who participated in an intervention study made dietary changes and that they also influenced the dietary habits of their family members. Children’s and spouses’ preferences influenced the success of these dietary changes. The biggest obstacle to maintaining a healthier lifestyle was socializing with their compatriots. Diet-related social norms mean that fatty and sweet food and sugary drinks are readily available [28]. Another study that focused on culturally adapted lifestyle education, including diet modification and increased physical activity among women from Pakistan, showed a change in intentions to alter the intake of selected foods. The intervention group’s daily intake of vegetables, fruit and juice increased. Culturally appropriate training tailored for Pakistani women jump-started their motivation to introduce healthier diets in small steps [29]. Furthermore, another study showed that a Chinese population that had moved to a Western context changed its diet. The majority of study participants reported awareness and knowledge of healthy eating with increased fruit and vegetable intake, as well as healthier cooking methods. But at the same time, portion size and the consumption of convenience foods increased. Therefore, it was important to adapt public health strategies to reinforce good dietary patterns while emphasizing the possible risk to health of consuming larger portion sizes and more convenience foods during immigration [30].

Participants in the present study found different strategies to control the number of meals they ate per day, the time interval between meals, and the amount and type of foods eaten. These strategies are in line with the Dietary Guidelines for Americans (DGA), which recommend a reduced intake of calories in the form of sugar and saturated fat [31]. Furthermore, to be able to control the contents of and composition of food, the best option is to cook at home. Homemade food is seen as healthy, and cooking at home should be encouraged [32]. Ideal weight is associated with status at all ages, and it can be decisive for social inclusion. This can be a significant factor in starting a weight loss programme, which, combined with self-motivation, can lead to lifestyle changes and a successful reduction in weight [33].

Obesity and physiological changes in the body during pregnancy may have psychosocial consequences related to negative body image [34, 35]. Psychological well-being is dependent on a positive body image. The foundation for body image is laid in childhood [36], indicating the importance of healthy lifestyle habits among mothers. Furthermore, our study shows that motivation to change one’s eating habits resulted in motivation to change family members’ eating habits to healthier ones. The biggest motivating factor was the desire to be a good role model for their children, prevent diseases such as diabetes and age healthily.

Our study shows that body image strongly influenced the degree to which participants felt empowered to tackle the problem of being overweight. Self-efficacy relative to body image and eating behaviour can promote acceptance of body diversity [37], healthy eating and physical activity [38].

### Limitations of the study

Overall, the limitations of the study include drawing participants from only one nutrition course in Oslo, only women who could read and write Norwegian were allowed to participate in the study, and the fact that the participants were not randomly selected, lowers the generality of the findings in the study. The sample may not be representative of the views of the broader population of immigrant women in Norway. However, the findings provide valuable insight into and understanding of immigrant women's self-reflections and attitudes towards their lifestyle and health. Another limitation of the study was that the SDM session was not recorded, however notes on the insight card were thoroughly taken. Nevertheless, the analysis may be less verifiable and increase the risk of bias.

## Conclusion

Motivation to change one’s eating habits and lifestyle to be healthy is fundamental to achieve change. The participants in our present study shared their strategies for dealing with unwanted weight gain. They all had a common strategy of searching for information on the Internet and actively participating in a nutrition course. In addition to that, the study participants also had to deal with differences between the cultural norms of their countries of origin and those they encountered in the Norwegian context. When motivated to change their eating habits, the study participants actively encouraged family members to do the same. Development of culture sensitive nutrition guidance is important for nutrition education and health promotion in an immigrant population. It is purposeful that the information is easily available both online and in health care settings. Knowledge from our study can be translated to similar contexts and can be a starting point for carrying out larger intervention studies.

## Data Availability

The collected data and personal information have been treated confidentially and in accordance with data protection regulations (GDPR). Presentation of the results will remain anonymous. It will not be possible for participants to be identified in the publication. The data supporting the findings of this study are available upon request from the corresponding author but cannot be made publicly available due to ethical concerns.

## Abbreviations

SDM: Story-dialogue method
KIA: Christian Intercultural Work
NNR: Nordic Nutrition Recommendations
Kg: Kilograms
DGA: The Dietary Guidelines for Americans
GDPR: Data protection regulations
SHWT: Sustainable health care and welfare technologies
